# Simultaneous and Rapid Determination of Main Lignans in Different Parts of *Schisandra Sphenanthera* by Micellar Electrokinetic Capillary Chromatography

**DOI:** 10.3390/molecules16053713

**Published:** 2011-05-03

**Authors:** Guangxin Yuan, Yang Liu, Tan Li, Yan Wang, Yu Sheng, Ming Guan

**Affiliations:** 1 School of Pharmaceutical Sciences, Jilin University, Changchun, 130021, China; 2 Pharmaceutical College of Beihua University, Jilin, 132013, China; 3 Life Scientific Research Center of Beihua University, Jilin, 132013, China

**Keywords:** micellar electrokinetic capillary chromatography (MEKC), lignans, different plant parts, *Schisandra sphenanthera*

## Abstract

Lignans are imporant active ingredients of *Schisandra sphenanthera*. A micellar electrokinetic chromatography method was developed for the simultaneous determination of eight lignans – schizandrin, schisandrol B, schisantherin A, schisanhenol, anwulignan, deoxyschizandrin, schizandrin B and schizandrin C – in different parts of *S. sphenanthera*. The key factors for separation and determination were studied and the best analysis conditions were obtained using a background electrolyte of 10 mM phosphate-37.5 mM SDS-35% v/v acetonitrile (pH 8.0) at the separation voltage of 28 kV and detection at 214 nm, whereby the plant samples could be analyzed within 9.0 min. Analysis yielded good reproducibility (RSD between 1.19-2.28%) and good recovery (between 92.2-103.8%). The detection limits (LOD) and limit of quantification (LOQ) were within 0.4-1.2 mg/L and 1.5-4.0 mg/L. This method is promising to improve the quality control of different parts of *S. sphenanthera*.

## 1. Introduction

*Schisandra sphenanthera* Rehd. et Wils (Schisandraceae) is a well-known medicinal plant in Traditional Chinese Medicine. The fruits and seeds have been used for centuries as an antitussive, tonic and sedative agent, and in traditional medicine to improve the liver function of patients with viral hepatitis [1]. In traditional Chinese medicine, the dried ripe fruits of both *Schisandra sphenanthera* and *Schisandra chinensis* have long been used as Wuweizi, even though their chemical constituents and contents of the bioactive components are quite different. Since 2000, they have been accepted as two different crude drugs, Nan-Wuweizi and Bei-Wuweizi, respectively, by the Chinese Pharmacopoeia [2,3].

**Figure 1:**
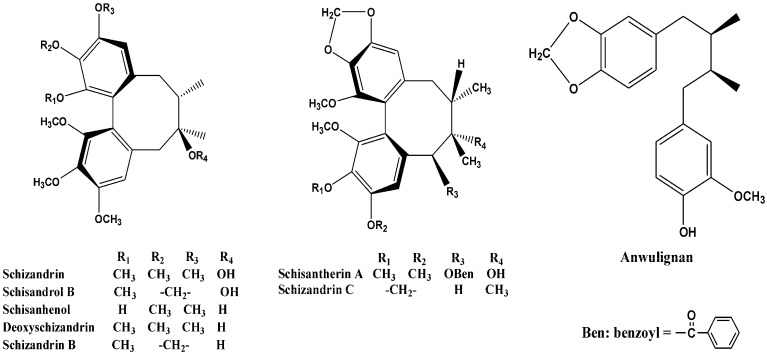
Chemical structures of the eight lignans.

Many studies have indicated that the active ingredients in *S. sphenanthera* are lignans possessing an unusual structure derived from dibenzo[a,c]cyclooctadiene [4]. These lignans can lower the serum glutamate-pyruvate transaminase (SGPT) level, inhibit platelet aggregation, and show antioxidative, calcium antagonizing, antitumor-promoting, and anti-HIV (human immunodeficiency virus) effects [5,6,7,8]. The content of lignans in *S. sphenanthera* is relatively low and is made up of at least 50 individual components. Moreover, the concentration of these lignan components varies considerably from plant to plant and has a genetic background, but eight compounds: schisandrin (**1**), schisandrol B (**2**), schisantherin A (**3**), schisanhenol (**4**), anwulignan (**5**), deoxyshisandrin (**6**), schisandrin B (**7**) and schisandrin C (**8**), represent the main lignan components of *S. sphenanthera* [9]. The chemical structures of these eight lignans are shown in Figure 1. 

So far, there are reports in the literature [10,11,12,13,14,15,16] about the simultaneous determination of the main components in *S. sphenanthera*, but few studies have been reported about some of the very important lignan components, such as schisanhenol and anwulignan. Recently, a high performance liquid chromatography with photodiode array detection (HPLC-DAD) method [17] has been developed and successfully applied to analyse and quantify eight bioactive components from friuts of *S. sphenanthera*. However, the method suffers from long analysis times and a high consumption of organic solvents.

Capillary electrophoresis (CE), with its high resolving power, low solvent consumption, and simple sample pretreatment is currently one of the most popular methods for separation and monitoring of Traditional Chinese Medicines (TCMs) [18,19,20,21]. To the best of our knowledge, there are no published CE methods for the eight lignans have been fully validated, including extraction recoveries, sample preparation, stability and analytical procedure. Morover, there is no systematic study on simultaneous determination of the eight lignans in different parts of *S. sphenanthera*. 

In the present study, a micellar electrokinetic capillary chromatography (MEKC) method has been developed for the simultaneous and rapid determination of eight key lignans in seed, pulp, stem, rattan and leaf parts of *S. sphenanthera*. The method should be useful for *S. sphenanthera* quality control applications.

## 2. Results and Discussion

### 2.1. Choice of Sample Treatment Methods

Solvent extraction (SE), ultrasonication extraction (UE), microwave extraction (ME), and pressure solvent extraction (PSE) are often used to extract lignans. All of tested extraction procedures provided high extraction efficiency under the optimal conditions. In this work, UE was selected to extract lignans. At the same time, different extraction solvents (water, methanol, acetonitrile, acetone, and chloroform) were investigated, and finally methanol was optimized to extract lignans from the *S. sphenanthera*. The results showed that recoveries for the main lignans were all over 92%.

### 2.2. Effect of Buffer Type and Concentration

To determine the optimum MEKC conditions, several buffers were investigated, including borate, phosphate, and ammonium/ammonia. The combined use of 20 mM phosphate-30 mM SDS-10% v/v methanol was found to be the most effective for resolving the lignan mixture. Although a good separation of the lignans was achieved, 36 min were required to separate them.

The effect of the ionic strength of the buffer on the separation of solution was studied next. The phosphate buffer concentrations were varied from 5 to 25 mM. Results showed that the lower concentrations of the buffer (5 mM) resulted in poor peak shape and resolution, while the higher concentrations (10–25 mM) resulted in improved resolution. On the other hand, the migration times and the peak width of all peaks were increased with the increase in the buffer concentration because the increase of the ionic strength can cause a lower zeta-potential on the capillary surface that would lead to the suppression of electroosmotic flow (EOF). A concentration of 10 mM phosphate buffer was finally chosen, because it afforded the best compromise between good peak shape, resolution and suitable analyte migration times.

### 2.3. Effect of SDS Concentration

The concentration of surfactant is an important condition affecting analysis time and selectivity. In this work the effect of SDS concentration was evaluated over the range from 27.5 to 42.5 mM. With increasing concentrations of SDS, longer migration times and higher currents were observed. When the SDS concentration exceeded 40.0 mM, the analysis time exceeded 30 min and peak shapes were asymmetric. When the SDS concentration was reduced, the peak shape gradually improved, but the resolution between schisanhenol, schizandrin B and impurity peaks was greatly decreased. With concurrent consideration in peak shape, resolutions and migration times of these analytes, SDS concentration was fixed at 37.5 mM for the subsequent experiments.

### 2.4. Effect of pH

The pH of the buffer has a pronounced effect on separation selectivity because it affects both charged species and the magnitude of EOF generated. Therefore, the manipulation of buffer pH is usually a key strategy to optimize a separation. In this study, the effect of buffer pH on the separation in the pH range of 7.0–8.5 (7.0, 7.5, 7.8, 8.0, 8.2 and 8.5) was investigated. Results showed that the migration times of these analytes decreased with increasing pH from 7.0 to 8.0 but the resolution remained approximately the same. However, if the buffer pH was higher than 8.0, peak width of these analytes increased, resulting in poor resolution. Considering the resolutions and migration times, pH 8.0 was chosen as the optimal condition.

### 2.5. Effect of Organic Modifier

Organic solvents, the most common of which are methanol, ethanol, and acetonitrile, are used as additives to micellar solutions. The organic modifier alters the polarity of the aqueous phase, the electrolyte viscosity and the zeta potential. Methanol, ethanol, and acetonitrile were added as modifiers to the buffer solutions in different percentages. The addition of methanol or ethanol increased the migration times of the analytes. On the other hand, the effect of acetonitrile concentration was studied in the range 10–40% v/v. The migration times and peak width of these analytes considerably decreased when the acetonitrile percentage in buffer increased. However, when the concentration of acetonitrile was more than 35%, impurity peaks between schisanhenol and schizandrin B overlapped partly with that of schisanhenol and schizandrin B. Considering the analysis time and resolution of impurity peaks, schisanhenol and schizandrin B, 35% v/v acetonitrile was identified as a good compromise for a rapid separation with high resolution.

### 2.6. Effect of the Applied Voltage

High voltage was required in CE to reduce the analysis time; voltages from 20 to 30 kV were investigated. It was found that analysis time decreased with increasing voltage but when the voltage was greater than 28 kV it could result in a high operating current which could easily lead to Joule heating problems. The best compromise in terms of resolution, current generated, and analysis time was found to be 28 kV.

### 2.7. Effect of Temperature

The effect of temperature on separation was investigated in the range of 20–30 °C. An increase in temperature resulted in decreased migration times and poor resolution. This phenomenon might result from the direct effect of increasing temperature on the rate of EOF and buffer depletion occurred more rapidly. Finally, 25 °C was selected as it gave the best compromise between resolution and run time with an acceptable level of the baseline noise. Altogether, according to all the previously mentioned results, the best resolution was obtained with a buffer solution containing 10 mM phosphate-37.5 mM SDS-35% v/v acetonitrile, and 28 kV applied voltage at 25 °C and schizandrin, gomisin A, schisantherin A, schisanhenol, anwulignan, deoxyschizandrin, schizandrin B and schizandrin C were all well separated within 9.0 min under these optimum conditions.

### 2.8. Analytical Parameters

For evaluation of the quantitative applicability of the method, different concentrations of standard solutions of the eight lignans were analyzed under the optimum separation conditions. The linearity between the peak area (Y) and the concentration (C, mg/mL) were investigated. The linear regression equations, correlation coefficients (r) and linearity ranges are shown in Table 1. The results indicated that high linearity between Y and C was attainable over the concentration range studied. The detection limits (LOD) and limit of quantification (LOQ) were the concentrations of a compound at which its signal-to-noise ratios (S/N) were detected as 3:1 and 10:1, respectively. The values are also given in Table 1. 

**Table 1:** Linearity, LOD, and LOQ of the eight lignan components.

Analyte	Regression equation	Correlation coefficient	Linear range (mg/L)	LOD (mg/L)	LOQ (mg/L)
Schisandrin	Y = 0.8057 X – 2.2907	0.9993	2.5-1000	0.5	2.0
Schisandrol B	Y = 0.5058 X – 12.357	0.9994	2.5-1000	1.0	3.0
Schisantherin A	Y = 0.4028 X – 7.5343	0.9991	2.0-800	0.8	2.5
Schisanhenol	Y = 0.2146 X + 17.367	0.9993	2.0-800	0.4	1.5
Anwulignan	Y = 0.2433 X – 6.779	0.9990	2.0-800	1.2	4.0
Deoxyschizandrin	Y = 0.3875 X + 6.3348	0.9996	2.5-1000	1.0	3.0
Schisandrin B	Y = 0.229 X + 12.245	0.9993	2.5-1000	1.0	3.0
Schisandrin C	Y = 0.1515 X + 21.072	0.9981	2.0-800	1.0	3.0

The seed of sample 1 (*S. sphenanthera* collected from Sichuan Province) was used to study the reproducibility of the method. The RSDs (n = 5) of the peak area and the migration time were 0.86% and 1.68% for schisandrin, 0.95% and 1.19% for schisandrol B, 1.31% and 1.79% for schisantherin A, 1.62% and 1.98% for schisanhenol, 1.91% and 2.04% for anwulignan, 2.59% and 1.83% for deoxyschizandrin, 2.33% and 1.80% for schisandrin B, 3.06% and 2.28% for schisandrin C, respectively. The recovery of the method was also examined by determining this sample. The overall recoveries (as described in the recovery section) at the lower addition amount (0.5 mg/g) and higher addition amount (2.5 mg/g) were 93.3% and 95.1% for schisandrin, 103.5% and 101.8% for schisandrol B, 94.4% and 97.5% for schisantherin A, 94.6% and 95.2% for schisanhenol, 1.0.4% and 103.8% for anwulignan, 95.3% and 96.8% for deoxyschizandrin, 102.9% and 100.4% for schisandrin B, 92.2% and 93.5% for schisandrin C, respectively. These data demonstrate that the developed MEKC method is reliable.

### 2.9. Application

**Figure 2:**
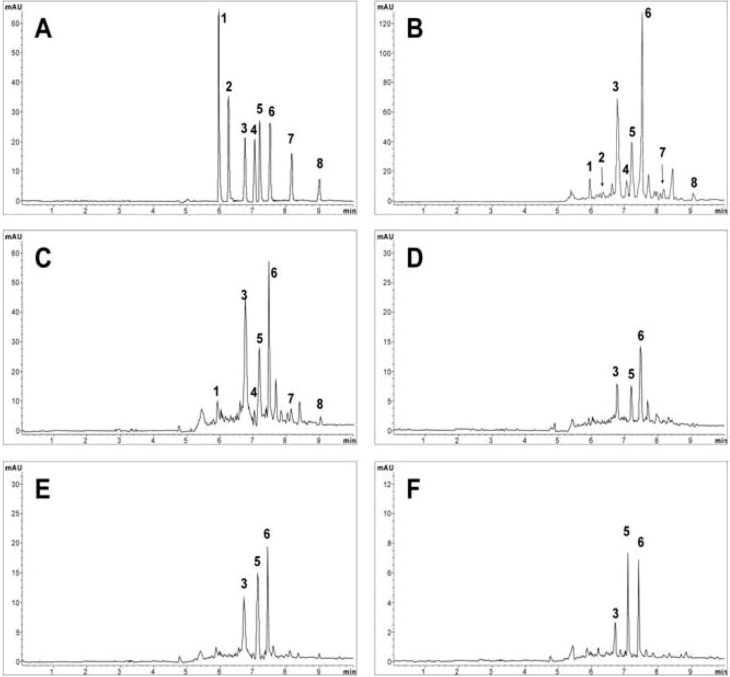
Typical electropherograms of standards and samples. (**A**) Standard mixture solutions, (**B**) Seed, (**C**) Pulp, (**D**) Stem, (**E**) Rattan, (**F**) Leaf. MEKC conditions: background electrolyte, 37.5 mM SDS and 35% v/v acetonitrile in 10 mM phosphate buffer (pH 8.0). Applied voltage was 28 kV. Capillary temperature: 25.0 °C. Detection: 214 nm. Peak identification: 1, schisandrin; 2, schisandrol B; 3, schisantherin A; 4, schisanhenol; 5, anwulignan; 6, deoxyschizandrin; 7, schisandrin B; 8, schisandrin C.

The contents (Table 2) of eight lignans in different parts of *S. sphenanthera* were determined using the developed MEKC method. The electropherograms of the standards and samples are shown in Figure 2. Peaks were identified by comparison of the migration times and by spiking the standards to the sample solution. From Figure 2, it can be seen that the eight lignans were well resolved within 9.0 min, and they can be separated from the other lignans or interferences.

**Table 2:** Content (mg/g, n = 3) of eight lignans in different parts of *Schisandra sphenanthera.*

Source	Parts	1	2	3	4	5	6	7	8
Sichuan	Seed	0.51	0.12	4.53	0.31	2.51	4.83	0.28	0.37
	Pulp	0.16	-^ b^	2.14	0.11	1.35	1.95	0.15	0.23
	Stem	-^ b^	-^ b^	0.39	-^ b^	0.36	0.57	-^ b^	+^ a^
	Rattan	-^ b^	-^ b^	0.45	+^ a^	0.48	0.68	-^ b^	+^ a^
	Leaf	-^ b^	-^ b^	0.18	-^ b^	0.25	0.29	-^ b^	-^ b^
Hunan	Seed	0.28	0.83	0.89	1.34	2.04	13.69	+^ a^	+^ a^
	Pulp	0.15	0.42	0.37	0.53	1.02	4.63	-^ b^	-^ b^
	Stem	+^ a^	0.32	0.12	0.28	0.35	1.22	-^ b^	-^ b^
	Rattan	+^ a^	0.36	0.16	0.31	0.42	1.52	-^ b^	-^ b^
	Leaf	-^ b^	0.17	+^ a^	0.11	0.14	0.63	-^ b^	-^ b^
Shanxi	Seed	-^ b^	-^ b^	6.5	0.65	5.63	8.4	0.31	0.76
	Pulp	-^ b^	-^ b^	3.13	0.30	2.21	3.9	-^ b^	+^ a^
	Stem	-^ b^	-^ b^	0.88	0.14	0.49	1.26	-^ b^	-^ b^
	Rattan	-^ b^	-^ b^	1.06	0.16	0.65	1.54	-^ b^	-^ b^
	Leaf	-^ b^	-^ b^	0.42	-^ b^	0.16	0.51	-^ b^	-^ b^

^a^ + : below LOQ; ^b^- : below LOD.

## 3. Experimental

### 3.1. Materials and Reagents

Standards of the compounds schizandrin, schisandrol B, schisantherin A, schisanhenol, anwulignan, deoxyschizandrin, schizandrin B and schizandrin C were purchased from the National Institute for the Control of Pharmaceutical and Bio-products of China (Beijing, China). Three raw material samples of *S. sphenanthera* were collected from different provinces in China. Sodium doecyl sulfate (SDS) was purchased from Genview (Houston, TX, USA). Methanol, ethanol, and acetonitrile were of HPLC grade and purchased from Guoyao Biotechnology Co. (Shanghai, China). Ammonium acetate, sodium borate, boric acid, disodium hydrogen phosphate and sodium dihydrogenphosphate were purchased from Guoyao Biotechnology Co. (Shanghai, China). Water was purified using a Milli-Q system (Millipore, Bedford, MA, USA). All the reagents were of analytical grade.

### 3.2. Apparatus and Method

Electrophoretic measurements were performed on an HP^3D^ capillary electrophoresis system (Agilent Technologies, Waldbronn, Germany) equipped with a diode-array detector, a thermostated capillary cartridge, a high-voltage built-in power supply, and an autosampler. The Agilent ChemStation software package (Agilent Technologies) was used for the acquisition and subsequent treatment of the electropherograms. The apparent pH value was measured with a pH meter (Shanghai Weiye Factory, Shanghai, China). The separations were carried out with a bare fused-silica capillary (Yongnian Photo Fiber Factory, Hebei Province, China) with an inner diameter of 50 µm and a length of 50 cm (41.5 cm to the detector window). Before its first use, the capillary was washed sequentially with 1.0 M NaOH for 10 min, 0.1 M NaOH for 20 min, distilled water for 5 min, and running buffer for 5 min. Among analyses, the capillary was flushed with 0.1 M NaOH for 3 min, distilled water for 3 min, and running buffer for 4 min in order to get good repeatability. When not in use, the capillary was stored in distilled water. The capillary voltage was +28 kV and the operating temperature 25 °C. Sample injection was performed by applying a pressure of 50 mbar for 3 s at the capillary inlet. The run buffer was 10 mM phosphate (pH 8.0)-37.5 mM SDS-acetonitrile (35% v/v). All solutions were filtered through a 0.45 µm filter before use.

### 3.3. Solutions and Sample Preparation

Stock solutions of schizandrin (1,000 µg/mL), gomisin A (1,000 µg/mL), schisantherin A (800 µg/mL), schisanhenol (800 µg/mL), anwulignan (800 µg/mL), deoxyschizandrin (1000 µg/mL), schizandrin B (1,000 µg/mL) and schizandrin C (800 µg/mL) were prepared in methanol. Analytical solutions were prepared from them by appropriate dilution with methanol. Prior to use, all solutions and run buffers were degassed by ultrasound for 10 min and filtered through a 0.45 µm membrane filter. The seeds, pulp, stems, rattan, and leaves of *S. sphenanthera* were dried to a constant weight at 60 °C in a vacuum oven, then pulverized to powder (about 40-mesh) with a disintegrator. Ten g of each dried powder was accurately weighed and mixed with methanol (300 mL), and then it was extracted under ultrasound for 20 min. After it was filtered, the residue was re-extracted with methanol twice more (150 mL each time) using the same procedure. The three filtrates were combined, evaporated to dryness under vacuum, and the residue was dissolved in methanol (100 mL). A sample of the methanol extract (500 µL) was evaporated to dryness under vacuum and dissolved in buffer solution (500 µL) under sonication for MEKC analysis.

### 3.4. Recovery

The efficiency for sample pretreatment was validated by a recovery investigation. In the initial step of the sample preparation, each standard of schizandrin, schisandrol B, schisantherin A, schisanhenol, anwulignan, deoxyschizandrin, schizandrin B and schizandrin C (1.0 and 5.0 mg) was added to of the dried powder of seed from sample 1 (*S. sphenanthera* collected from Sichuan Province, 2.0 g). They were then treated as described in the previous section (solutions and sample preparation). Finally, the prepared sample solution was analyzed using the developed MEKC method, and the recovery was determined.

## 4. Conclusions

A simple, rapid and sensitive MEKC method has been developed for the simultaneous quantitative analysis of eight lignans in the seed, pulp, stem, rattan and leaf parts of *S. sphenanthera*. To our best knowledge, this analysis may be the first example involving the simultaneous detection of the eight lignans in *S. sphenanthera* by the MEKC method. This method showed good linearity, sensitivity and sufficient limit of detection. It was evident that this approach was a useful and rapid technique for identification and determination of main lignans in *S. sphenanthera*. The results indicate that differences in the content of the eight lignans among the different parts of *S. sphenanthera* are great, being highest in the seed and lowest in the leaf. The evaluation of data could be useful for screening the optimal condition of extraction of different part of *S. sphenanthera*.
